# *GPX3* rs8177412 Polymorphism Modifies Risk of Upper Urothelial Tumors in Patients with Balkan Endemic Nephropathy

**DOI:** 10.3390/medicina59081421

**Published:** 2023-08-04

**Authors:** Zana Radic Savic, Vesna Coric, Stojko Vidovic, Vanja Vidovic, Jelena Becarevic, Irina Milovac, Zorica Reljic, Bosa Mirjanic-Azaric, Ranko Skrbic, Radoslav Gajanin, Marija Matic, Tatjana Simic

**Affiliations:** 1 Department of Medical Biochemistry, Faculty of Medicine, University of Banja Luka, 78000 Banja Luka, Bosnia and Herzegovina; zana.radic@med.unibl.org (Z.R.S.); bosa.mirjanic@med.unibl.org (B.M.-A.); 2 Centre for Biomedical Research, Faculty of Medicine, University of Banja Luka, 78000 Banja Luka, Bosnia and Herzegovina; stojko.vidovic@med.unibl.org (S.V.); vanja.vidovic@med.unibl.org (V.V.); jelena.becarevic@med.unibl.org (J.B.); irina.milovac@med.unibl.org (I.M.); 3 Institute of Medical and Clinical Biochemistry, Faculty of Medicine, University of Belgrade, 11000 Belgrade, Serbia; vesna.coric@med.bg.ac.rs; 4 Center of Excellence for Redox Medicine, Biotech Place, 2W-017, 575 North Patterson Avenue, Winston-Salem, NC 27157, USA; 5 Department of Human Genetics, Faculty of Medicine, University of Banja Luka, 78000 Banja Luka, Bosnia and Herzegovina; 6 Medical Laboratory “PAN LAB”, 36000 Kraljevo, Serbia; zorica.reljic@gmail.com; 7 Department of Pharmacology, Toxicology and Clinical Pharmacology, Faculty of Medicine, University of Banja Luka, 78000 Banja Luka, Bosnia and Herzegovina; ranko.skrbic@med.unibl.org; 8 Academy of Sciences and Arts of the Republic of Srpska, 78000 Banja Luka, The Republic of Srpska, Bosnia and Herzegovina; 9 Department of Pathological Anatomy, Faculty of Medicine, University of Banja Luka, 78000 Banja Luka, Bosnia and Herzegovina; radoslav.gajanin@med.unibl.org; 10 Serbian Academy of Sciences and Arts, 11000 Belgrade, Serbia

**Keywords:** Balkan endemic nephropathy, upper tract urothelial cell carcinoma, aristolochic acid, *GPX3* polymorphism

## Abstract

Current data suggest that aristolochic acid (AA) exposure is a putative cause of Balkan endemic nephropathy (BEN), a chronic kidney disease strongly associated with upper tract urothelial carcinoma. The cellular metabolism of AA is associated with the production of reactive oxygen species, resulting in oxidative distress. *Purpose:* Therefore, the aim of this study was to analyze individual, combined and cumulative effect of antioxidant gene polymorphisms (*Nrf2* rs6721961, *KEAP1* rs1048290, *GSTP1AB* rs1695, *GSTP1CD* rs1138272, *GPX3* rs8177412 and *MDR1* rs1045642), as well as *GSTP1ABCD* haplotypes with the risk for BEN development and associated urothelial cell carcinoma in 209 BEN patients and 140 controls from endemic areas. *Experimental method:* Genotyping was performed using polymerase chain reaction (PCR) and PCR with confronting two-pair primers (PCR-CTTP) methods. *Results:* We found that female patients carrying both variant *GPX3* rs8177412 and *MDR1* rs1045642 genotypes in combination exhibited significant risk towards BEN (OR 1 = 3.34, 95% CI = 1.16–9.60, *p* = 0.025; OR 2 = 3.79, 95% CI = 1.27–11.24, *p* = 0.016). Moreover, significant association was determined between *GPX3*rs8174412 polymorphism and risk for urothelial carcinoma. Carriers of variant GPX3*TC + CC genotype were at eight-fold increased risk of BEN-associated urothelial tumors development. There was no individual or combined impact on BEN development and BEN-associated tumors among all examined polymorphisms. The haplotype consisting of variant alleles for both polymorphisms G and T was associated with 1.6-fold increased risk although statistically insignificant (OR = 1.64; 95% CI = 0.75–3.58; *p* = 0.21). *Conclusions:* Regarding *GPX3* rs8177412 polymorphism, the gene variant that confers lower expression is associated with significant increase in upper urothelial carcinoma risk. Therefore, BEN patients carrying variant *GPX3* genotype should be more frequently monitored for possible upper tract urothelial carcinoma development.

## 1. Introduction

Balkan endemic nephropathy (BEN) is recognized as a familial, slowly progressive, tubulointerstitial kidney disease that indubitably leads to end-stage renal disease (ESRD) [1,2]. This endemic disease occurs in the agrarian communities of Serbia, Bosnia, Bulgaria and Croatia on the Danube River and its tributaries [3,4]. One of the most important hallmarks of BEN is its strong association with upper urinary tract urothelial carcinoma [3]. Furthermore, earlier data indicate that patients suffering from BEN have, even up to 100 times, a higher prevalence of urothelial tumors.

Although several theories in the past were proposed to explain BEN causality, exposure to aristolochic acid (AA) has been adopted as the primary causative agent in BEN, particularly concerning the development of BEN-associated carcinoma [2]. New evidence suggest that some crops can bioaccumulate AA from soil and water. Likewise, it is believed that patients from endemic areas could be exposed to AA by ingesting food prepared from contaminated flour [5]. Additionally, a number of evidence indicate the causality relationship between BEN and Chinese herb nephropathy (CHN). According to a study by De Broo et al., CHN and BEN are two entities of one widespread disease called aristolochic acid nephropathy (AAN), based on the clinical and pathohistological similarities of both diseases [6].

Considering oxidative and reductive route of AA metabolism, numerous enzymes and coenzymes participate in activation and detoxication of AA, which leads to excessive generation of reactive oxygen species (ROS) [7,8]. AA exposure is associated with mitochondrial dysfunction, enhanced ROS production, impaired mitochondrial membrane potential and reduction in ATP production [9,10]. Furthermore, during the bioactivation of AA genotoxic metabolite N-hydroxyaristolactam I is formed [11]. Both disruption of redox homeostasis and N-hydroxyaristolactam lead to harmful effects on vital macromolecules, including DNA, which result in mutagenesis and carcinogenesis [12]. Thus, high mutagenic N-hydroxyaristolactam I covalent adducts with DNA and characteristic A→T transversions were detected in malignantly altered kidney tissue obtained from patients with BEN [13]. Therefore, AA-induced oxidative stress and AA-elicited genotoxicity might explain the high frequency of tumors, as well as renal damage in BEN [8,12]. 

Oxidative stress may be at least partially dependent on altered Keap1/Nrf2 (Kelch-like ECH-associated protein 1/nuclear factor, erythroid 2-like transcription factor 2) signaling pathway [14]. In order to prevent oxidative stress, several intracellular and extracellular antioxidant systems, including enzymatic glutathione S-transferases (GSTs) and glutathione peroxidases (GPX), are enrolled. In addition, genetic polymorphisms described in genes encoding all these proteins result in reduced or altered activity of these enzymes and thus the ability to neutralize ROS. Despite convincing data in favor of the disturbance of redox homeostasis in the AA metabolism, it is still unclear whether free radicals are the key molecular mediators in the pathobiology of BEN and associated urothelial carcinoma. Useful approach to study the role of free radicals in the pathophysiological mechanisms of BEN is analysis of the role of genetic polymorphisms of antioxidant enzymes in the susceptibility to this disease. Previous attempts to decipher the role of oxidative stress in the pathogenesis of BEN showed that polymorphisms implicated in the antioxidative protection contribute to BEN development [15,16]. Nrf2, the master regulator of redox homeostasis, in physiological conditions, is located in the cytoplasm within an inactive complex bound to Keap1, which is responsible for the continuous ubiquitylation and degradation of Nrf2 [14]. Concisely, Neh2 domain of Nrf2 interacts with Keap1 through two motifs: ETGE and DGR domain [14,17]. Exposure to xenobiotic stressors or endogenous disturbances results in the dissociation of Nrf2 from Keap1, and its translocation to the nucleus, where it binds to the antioxidant response elements (AREs), located in the promoter region of antioxidant and phase II detoxifying target genes [17]. The most studied *Nrf2* single nucleotide polymorphism (SNP) is *Nrf2* rs6721961 (*c.617 C* > *A*), located in the ARE-like motif, characterized by a *C* > *A* substitution. *Nrf2* rs6721961 leads to reduced basal expression of Nrf2 and affects binding to AREs [18,19]. On the other hand, *KEAP1* rs1048290 (*c.1413 C* > *G*) SNP, marked by *C* > *G* substitution, is located in the genomic region encoding DGR domain, which disrupts binding of Nrf2 and Keap1 [20]. It might result in increase of Keap1 protein expression level [20,21], constitutive stabilization and cytoplasmic accumulation of Nrf2 compromising the antioxidant response [22]. Another important Nrf2-targeted genes are cytosolic members of glutathione transferases (GSTs), which represent first-line enzymatic antioxidant protection and belong to phase II family of enzymes involved in the detoxification of various toxic compounds. Expression of genes of GST enzyme superfamily members, including GSTP1, is tightly regulated by transcription factor Nrf2 [23]. Kidneys exhibit high expression of cytosolic GSTs, especially the *pi* (GSTP) isoenzymes. In the case of *GSTP1* gene polymorphisms, two most commonly occurring SNPs are rs1695 and rs1138272. Regarding *GSTP1*rs1695, this polymorphism results in amino acid substitution of isoleucine with valine at position 105 (Ala105Val), whereas the presence of T instead of C at position 341 results in coding of protein with valine instead of alanine (rs1138272, Ala114Val). Both amino acid changes lead to decreased enzyme activity or modified substrate specificity [23]. These two polymorphisms in combination represent haplotype *GSTP1ABCD*. Furthermore, among various proteins encoded by *Nrf2*-targeted genes, there are also several ATP binding cassette (ABC) transporters, including multidrug resistance protein 1 (MDR1) [24,25]. As a part of the Phase III detoxification system, MDR1 functions as an ATP-dependent exporter of various xenobiotics from cells [26]. Regarding *MDR1* polymorphism, *MDR1* rs1045642 (*c.3435 C* > *T*) SNP, the first described polymorphism among all *MDR1* SNPs with altered protein expression, results in lower MDR1 (P-glycoprotein) expression in the kidneys [26]. Notably, there is experimental evidence showing that GSTs and members of ABC transporters could be involved in AA biotransformation [27,28]. It is noteworthy to mention that polymorphism of another key antioxidant enzyme glutathione peroxidase 3 (GPX3) has not been studied in context of BEN as yet. GPX3 belongs to family selenium-dependent peroxidases and plays a pivotal role in decreasing extracellular oxidative damage by reducing hydrogen peroxide and organic hydroperoxides to water. GPX3 is mostly a plasma enzyme and around 70% of GPX3 is secreted by the basolateral membrane of kidney proximal tubule [29]. Polymorphism in the gene encoding *GPX3 (rs8177412)* is responsible for the downregulation of gene transcription, resulting in markedly decreased plasma activity of the GPX3 [30].

Although the results obtained in animal models of AA toxicity suggest the essential role of oxidative stress, its role in the pathophysiological mechanism of BEN and BEN-associated tumors has not been discerned in these patients, in contrast to ESRD [31,32]. Interestingly, there are individual differences among individuals exposed to AA. The possible explanation for these dissimilar responses may be inter-individual differences in the activities of enzymes involved in defense against oxidative stress and/or biotransformation of AA. Therefore, the aim of the study was to investigate whether individual susceptibility towards BEN may rely on functional variations of genes encoding antioxidant regulatory and catalytic proteins. Particular emphasis was given to upper tract urothelial carcinoma, as the most prominent feature in BEN patients. To clarify the activity of endogenous mechanisms of antioxidant protection in BEN patients and BEN-associated tumors, we conducted a study with 209 BEN patients, with and without verified urothelial tumors, and 140 healthy controls from endemic regions.

## 2. Materials and Methods

### 2.1. Study Participants

The case-control study comprising 209 BEN patients and 140 sex- and age-matched controls was performed. BEN patients were selected at two dialysis centers in the Republic of Srpska, Bosnia and Herzegovina (endemic cities of Bijeljina and Šamac). All collected blood samples in the patient group were part of the DNA biobank of the Institute of Medical and Clinical Biochemistry, Faculty of Medicine, University of Belgrade (Serbia). Furthermore, diagnosis BEN was confirmed by clinical, laboratory and echosonographical examination using previously established criteria [2]. The control group included healthy volunteers from endemic areas with no family history for BEN. The inclusion criteria for the control group were as follows: normal kidney function verified by the blood levels of urea and creatinine, verified absence of hypertension, diabetes mellitus and malignancies, and being able to provide informed consent. The exclusion criteria for both groups comprised the presence of other malignant diseases and patients unwilling to participate. A structured questionnaire was used to collect the data regarding basic demographic information.

### 2.2. DNA Isolation

Genomic DNA was extracted from 200 µL EDTA-anticoagulated peripheral blood using PureLink^®^ gDNA Blood Kit (Invitrogen, Carlsbad, CA, USA; cat. No. K182001). Isolated DNA was stored at −20 °C until polymerase chain reaction (PCR) was performed. DNA samples from the biobank were isolated using the same method.

### 2.3. Genotyping

*Nrf2* rs6721961 polymorphism analysis was performed using polymerase chain reaction with confronting two-pair primers (PCR-CTPP) method according to the modified method [19]. The PCR reaction was performed on ProFlex PCR System (Applied Biosystems, Foster City, CA, USA). Amplified products were separated on 2% agarose gel (cat. No. G521802), and visualization of PCR products was enabled with E-Gel Power Electrophoresis System (Invitrogen, Carlsbad, CA, USA). As a *CC* referent genotype was considered the band with 282 bp and 113 bp. The band with 282 bp, 113 bp and 205 bp was considered as a *CA* heterozygous genotype, whereas the band with 205 bp and 113 bp was examined as a *AA* homozygous genotype.

To determine genotypes of *KEAP1* (rs1048290), *GSTP1AB* (rs1695), *GSTPA1CD* (rs1138272), *GPX3* (rs8177412) and *MDR1* (rs1045642) real-time PCR (qPCR) was performed on Applied Biosystems™ 7500 Real-Time PCR (Applied Biosystems, Foster City, CA, USA). TaqMan^®^ drug metabolism genotyping assays were used (C_9323035_1, C_3237198_20, C_1049615_20, C__25964717_20 and C_7586657_20) according to the instructions (Applied Biosystems, Foster City, CA, USA). The amplification reaction of the DNA segment consisted of 40 repeated cycles through four steps of denaturation including initial denaturation 95 °C for 10 min, denaturation at 95 °C for 15 s, and annealing and extension at 60 °C for 90 s. Results of genotyping were visualized by Applied Biosystems 7500 software v2.0.6 (Applied Biosystems, Foster City, CA, USA).

### 2.4. Statistical Analysis

Statistical Package for the Social Sciences (SPSS software version 17, SPSS Inc, USA) was used for statistical analysis. Continuous variables were expressed as mean ± standard deviation (SD) or median (minimum–maximum), depending on data distribution. Categorical variables were presented as frequency (*n*, %) counts. Comparison of categorical variables was performed using χ2 test. χ2 test was also used to test deviation of the genotype distribution from Hardy–Weinberg equilibrium for each polymorphism, in patients and the controls individually. The effect of genotypes on BEN risk was evaluated by logistic regression analysis and expressed by odds ratios (OR) and 95% confidence intervals (CI). OR was adjusted by age and gender. Multiple risk models were used to inspect mutually the effect of different genes, alone or combined contributory factors, on BEN. Determination of linkage disequilibrium (LD) between SNPs and haplotype analysis was performed using Haploview and SNPStats [33,34]. The LD strength was expressed as D′. *p* value of ≤0.05 was considered to be statistically significant.

## 3. Results

Demographic characteristics of 209 BEN patients and 140 controls are presented in Table 1. The average age of BEN group comprising 177 men and 92 women was 71.67 ± 6.54 years, whereas, in the control group, which includes 71 men and 69 women, the average age was 70.29 ± 6.94 years. As shown, there was no significant difference in both groups regarding gender, age and smoking. 

Genotype distributions of gene polymorphisms for regulatory and catalytic antioxidant proteins in BEN patients and the control group and the risk for BEN development are presented in Table 2. All genotypes in the control group were in the Hardy–Weinberg equilibrium (*KEAP1*: X^2^ = 0.695, *p* = 0.512; *Nrf2* X^2^ = 0.009; *p* = 0.921; *GSTPAB* X^2^ = 0.432, *p* = 0.118; *GSTPCD* X^2^ = 0.234, *p* = 0.628; *GPX3* X^2^ = 0.120; *p* = 0.728; *MDR1* X^2^ = 0.028; *p* = 0.866). Four risk models were evaluated. Model 1 with no adjustments, model 2 with other genes as covariates, model 3 with age and gender as confounding factors and model 4 with all previously combined factors. Regarding *KEAP1* polymorphism, no significant difference between genotype distribution among BEN patients and controls was revealed. Moreover, the frequency of *KEAP1* genotypes with at least one copy of variant KEAP1*G allele (KEAP1*CG*GG genotype) was the same among patients and controls (84%). Likewise, for subjects carrying *C/A and *A/A Nrf2 genotypes associated with lower transcription of Nrf2, no significant BEN risk was obtained across all examined models (OR 1 = 0.88, 95% CI = 0.55–1.46, *p* = 0.669; OR 2 = 0.91, 95% CI = 0.55–1.50, *p* = 0.723; OR 3 = 0.89, 95% CI = 0.54–1.46, *p* = 0.648; OR 4 = 0.83, 95% CI = 0.48–1.42, *p* = 0.608). Similarly, we found no independent influence of *GSTP1AB* rs1695 and *GSTP1CD* rs1138272 polymorphism on the risk of BEN. Furthermore, the two other investigated polymorphisms of *GPX3* and *MDR1* did not significantly influence the risk for BEN development, although a slight increase in odds ratio was noted, namely, regarding *GPX3* polymorphism, we observed that carriers of at least one copy of variant GPX3*C allele (GPX3*TC*CC genotype) were at moderately increased susceptibility towards BEN development. However, statistical significance was lacking across all four models (OR 1 = 1.25, 95% CI = 0.74–2.12, *p* = 0.389; OR 2 = 1.24, 95% CI = 0.72–2.11, *p* = 0.415; OR 3 = 1.26, 95% CI = 0.75–2.14, *p* = 0.374; OR 4 = 1.26, 95% CI = 0.74–2.15, *p* = 0.392). Regarding the polymorphism in *MDR1* gene, the results presented herewith show that the variant homozygotes MDR1*TT were at 1.8-fold increased risk of developing BEN. Still, these results were only near the threshold of statistical significance (adjusted OR 3 = 1.82, 95% CI = 0.97–3.40, *p* = 0.059).

In Table 3 are presented polymorphisms of antioxidant regulatory and catalytic genes (*KEAP1*, *Nrf2*, *GSTPAB*, *GSTPCD*, *GPX3* and *MDR1*) in combination. When *GPX3* and *MDR1*-variant genotypes were combined a certain statistically insignificant risk on BEN development was observed (OR 1 = 1.75, 95% CI = 0.86–3.55, *p* = 0.117; OR 2 = 1.86, 95% CI = 0.91–3.80, *p* = 0.087). Logistic regression analysis showed no substantial risk when *Nrf2* genotypes were analyzed in combination with *GSTP1AB*, *GSTP1CD*, *MDR1* and *KEAP1* genotypes.

In the next step, we focused on the evaluation of the potential combined effect of polymorphisms of *KEAP1*, *Nrf2*, *GSTPAB*, *GSTPCD*, *GPX3* and *MDR1* gene with respect to gender. The performed gender-stratification analysis did not obtain any significant risk association when the individual genotypes were assessed (Supplementary Tables S1 and S2). Although in the overall analysis, the combined *GPX3* and *MDR1* polymorphisms did not show statistically significant association with the risk of BEN development, this combination had a great impact on the female population. Furthermore, female patients who carried both *GPX3* and *MDR1* variant genotypes (GPX3*TC*CC genotype and MDR1*CT*TT genotype) were at a higher risk of BEN development (OR 1 = 3.34, 95% CI = 1.16–9.60, *p* = 0.025), which was confirmed in the adjusted model (OR 2 = 3.79, 95% CI = 1.27–11.24, *p* = 0.016) (Table 4). Interestingly, we did not observe a statistically significant association of this combination with the risk of BEN development in males (Table 5).

We further analyzed the effects of *GSTP1* rs1695 and rs1138272 polymorphisms that was performed using haplotype analysis. Effects of *GSTP1* rs1695 and rs 1138272 are presented in Table 6. Since both *GSTP1AB* and *GSTP1CD* polymorphisms are located on the same chromosome, we estimated the linkage disequilibrium (LD) between *GSTP1* polymorphisms, namely, we evaluated the nonrandom association of *GSTP1* alleles and expressed it as a normalized coefficient of LD (D′). We found a D′ of 0.647 between *GSTP1AB* rs1695 and *GSTP1CD* rs1138272, confirming a high LD between these SNPs.

As indicated in Table 7, the most prevalent haplotype among BEN patients (68%) and controls (70%) is GSTP1A, consisting of *A and *C wild-type alleles. Haplotype GSTP1C consisting of variant alleles of both polymorphisms *G and *T had the lowest frequencies in both patients and controls. Regarding the effect of the *GSTP1ABCD* haplotypes on BEN susceptibility, the haplotype consisting of variant alleles *G and *T was associated with 1.6-fold increased risk although statistically insignificant (OR = 1.64, 95% CI = 0.75–3.58, *p* = 0.210).

Apart from assessing the combined effect of analyzed genotypes, we examined whether the cumulative risk allele number is associated with BEN development (Table 8). According to the identified genotypes associated with modifying role in terms of BEN risk (Table 2), we added them up and observed that the cumulative effect of the combination of polymorphisms encoding for regulatory and catalytic antioxidant proteins contributed successively increasing the risk of endemic nephropathy with a growth cumulative index number, although none of the differences reached statistical significance. 

In our study, 32 BEN patients (15%) developed urothelial carcinoma. Bearing in mind that the incidence of urothelial carcinomas is much higher in BEN patients than in the general population, we further assessed the effects of the analyzed genotypes by performing a small case-only study on the patient’s group with BEN in terms of assessing the risk for urothelial carcinoma development in such population. The distributions of gene polymorphisms for regulatory and catalytic antioxidant proteins in BEN patients with upper tract urothelial tumors are summarized in Table 9. The results of our case-only study showed that a significant difference was observed regarding the distribution of *GPX3* genotypes. Moreover, the carriers of at least one variant GPX3*C allele (GPX3*TC*CC genotype) were at eight-fold increased risk of developing upper tract urothelial tumor compared to the carriers of the referent GPX3*TT genotype (OR = 8.16, 95% CI = 3.60–18.40, *p* = 0.001), which was also confirmed after the adjustment (OR = 8.48, 95% CI = 3.60–19.30, *p* = 0.001). Furthermore, *GSTP1CD* genotype consisting of at least one variant GSTP1CD*T allele, in the case of rs1138272, exhibited increased susceptibility towards urothelial carcinoma development in comparison to *GSTP1CD* wild-type homozygotes (OR 1 =1.71, 95% CI = 0.59–5.11, *p* = 0.312; OR 2 = 1.84, 95% CI = 0.61–5–53, *p* = 0.275) although the statistical significance was not reached. 

## 4. Discussion

This study presents comprehensive analysis of the significance of antioxidant gene polymorphisms (*Nrf2* rs6721961, *KEAP1* rs1048290, *GSTP1AB* rs1695, *GSTP1CD* rs1138272, *GPX3* rs8177412 and *MDR1* rs1045642) in BEN development and associated urothelial cell carcinoma. Moreover, haplotype analysis of *GSTP1ABCD* polymorphism was also performed. Among the six examined polymorphisms, there was no statistically significant impact of these polymorphisms on the susceptibility toward BEN development, individually or in combination. However, when the combined effect of the assessed polymorphisms was analyzed with respect to gender, female patients carrying both variant *GPX3* rs8177412 and *MDR1* rs1045642 genotypes in combination exhibited significant risk towards BEN development. Still, the haplotype *GSTP1*analysis also did not reach statistical significance, although the haplotype consisting of both variant alleles was associated with 1.6-fold increased risk. However, when assessing the cumulative effect of five different risk-associated genotypes an ascending trend of BEN risk was observed. The final step in our analysis included the association of these polymorphisms with the risk of BEN-related urothelial carcinoma. We found that variant GPX3*TC + CC genotype was independently associated with upper urothelial carcinoma risk. Furthermore, the data obtained showed that individuals carrying variant GPX3*TC + CC exhibit eight-fold higher risk of upper urinary tract tumors.

It is important to highlight that polymorphisms encoding regulatory and catalytic antioxidant proteins might be among the potential factors affecting the individual’s susceptibility to BEN [7]. Indeed, one of the challenges to decipher an individual’s susceptibility to BEN and its strong linkage with urothelial carcinoma may be the evaluation of enzymes involved in AA biotransformation and their genetic polymorphisms. AA, a widespread natural extract of the *Aristolochiaceae clematis* plants, is found in soil, corn and wheat grain from endemic villages [2,5]. According to its genotoxicity and association with urothelial cancers, AA was classified as a carcinogen by the International Agency for Research on Cancer (IARC) [2]. As previously described, throughout the metabolic biotransformation of AA, both in vivo and in vitro studies reported excessive generation of ROS [8,9,12,27,35]. In vitro studies demonstrated an increase in ROS and H2O2 levels, a decrease in glutathione (GSH) and a diminished activity of GPX, along with a reduced intra-renal antioxidant capacity [10,27,35]. Similar results obtained in in vivo studies confirmed that AA, besides aggravating oxidative stress, also impaired antioxidant enzymes activity, including GPX, and leads to mitochondrial dysfunction in a rat model of AAN, which suggests that oxidative stress could be an important piece of the complex puzzle of BEN etiology [9,36]. Moreover, several studies emphasized that treatment of the cells with antioxidants showed cytoprotective effects by reducing AA-induced ROS and genotoxicity. Indeed, treatment with N-acetyl cysteine (NAC) and GSH mitigated the nephrotoxic effect of AA in vitro [36,37,38].

Importantly, AA can lead to the alteration of regulatory proteins involved in oxidative stress, such as Nrf2 and Keap1 protein [35,36]. Precisely, AA increases expression level of Keap1 and diminishes Nrf2 protein expression, leading to disrupted expression of a broad range of protective, antioxidant and detoxifying proteins [36]. The position of *Nrf2* rs6721961 (*−617 C* > *A*) SNP in the middle of the ARE motif affects its binding to the ARE; therefore, carriers of the Nrf2*AA genotype express a reduced level of mRNA expression for numerous antioxidant enzymes [14,18]. To the best of our knowledge, this is the first study that assessed the effect of these polymorphisms on the risk of development of BEN and upper tract urothelial carcinoma. Surprisingly, the results of this study did not show an association between the polymorphic expression of *Nrf2* rs6721961 and *KEAP1* rs1048290 in the development of BEN. Similar results were obtained with respect to urinary tract tumors. Furthermore, we found no independent influence of *Nrf2* rs6721961 and *KEAP1* rs1048290 polymorphisms on the risk of BEN-associated urothelial tumors. Study of Reszka et al. also did not find any association between these SNPs and the risk of urinary bladder tumors [39].

Interestingly, a previous research demonstrated that aristolochyl-lactam nitrogen ion, the nitro product of AA, can be detoxified with GSH [28] in reaction most probably catalyzed by phase II enzymes, such as GSTP1. The role of genetic polymorphism of several GSTs classes has been investigated in BEN patients [15,40]. According to Reljic et al., the carriers of the variant GSTA1*B allele had an increased risk of BEN development compared to carriers of referent GSTA1*A/*A genotype [15]. In addition, one study in Bulgarian cohort of BEN patients reported that the active *GSTM1* genotype is more common in BEN patients, compared to the control group [41]. On the other hand, several studies evidenced that certain polymorphisms occurring within the *GSTP1* gene, modulate the risk of developing ESRD and BEN-associated carcinoma [31,40]. To date, only one study has assessed the polymorphic expression of *GSTP1AB* rs1695 and did not identify an association of this polymorphism with BEN and urothelial carcinoma risk [15]. The finding in our study is in accordance with this study, concluding that *GSTP1AB* rs1695 did not influence the risk for BEN development [15]. Contrarily, the role of *GSTP1CD* rs1138272 SNP and haplotype analysis *GSTP1ABCD* has not yet been studied in relation to susceptibility to BEN and BEN-associated urothelial tumors. Likewise, when assessing the potential value of *GSTP1CD* rs1138272 polymorphism in BEN patients, our results showed that GSTP1CD*CT*TT variant genotype did not have an impact on BEN development and urothelial carcinoma. Although *GSTP1ABCD* haplotype analysis revealed higher risk of BEN in carriers of both variant alleles, the observed effect did not reach statistical significance. Hopefully, further genotyping of a larger study could potentially provide a significant association of haplotype with increased risk for BEN development and therefore identify individuals who are candidates for an earlier screening.

It is important to note that phase III of metabolism is also involved in detoxification of AA; thus, polymorphism of enzymes involved in this phase may also influence BEN and upper tract urothelial tumor risk. The influence of *MDR1* rs1050450 SNP on the BEN risk has been recently studied [42]. As the polymorphism affects the expression and activity of this protein [26], the results of our study show that there is an increased risk for BEN development, although the result did not reach statistical significance. Our results are consistent with the study by Atanasova et al. that showed that the polymorphisms in the *MDR1* gene were not associated with an increased risk for developing BEN [42]. We believe that more extensive research with larger patient cohort is needed to elucidate the role of *MDR1* polymorphism in BEN risk.

Due to the fact that GPX3 is primarily expressed in kidney tissue and plays a role in the initiation and progression phase of renal carcinogenesis [29], we also analyzed the polymorphism of this important antioxidant enzyme in terms of BEN and BEN-associated urothelial carcinoma risk. In terms of BEN, we observed that carriers of at least one copy of variant GPX3*C allele (GPX3*TC*CC genotype) were at moderately increased risk of BEN development. On the other hand, when assessing *GPX3* rs8177412 polymorphism in female BEN patients, our results showed that GPX3*T/C and GPX3*C/C variant genotypes had an impact on BEN risk after being combined with *MDR1* variant genotypes. In addition, we found significant influence of *GPX3* rs8177412 polymorphism on the risk of BEN-associated upper tract urothelial tumors. The result showed that the GPX3*TC + CC variant is a significant risk factor for the development of BEN-associated carcinoma. Regarding the functional relevance of this polymorphism, it seems that alterations in the GPX3 promoter region modify the enzyme expression [30]. Therefore, our results are biologically plausible since the polymorphism of *GPX3* results in lower transcriptional activity and reduced intracellular expression of the GPX3 enzyme, which impairs catalytic GPX3 enzyme activity [29,43]. Moreover, our finding is in line with several studies confirming a lower expression of GPX3 in the tumor tissue. Namely, analysis of data in TCGA and GTEx databases [44,45] revealed that *GPX3* gene expression, associated with several tumor types, was higher in normal bladder tissue than in bladder carcinoma tissue [46]. Integrated pan-cancer analysis discussed that GPX3 was under expressed in 22 of 34 examined tumor tissue samples, including urothelial bladder carcinoma [47]. Notably, latest research proposed GPX3 as a novel urine biomarker, based on relative expressions of *GPX3*. Moreover, GPX3 level was significantly lower in urine of patients with bladder carcinoma than in controls [46]. Ultimately, *GPX3* rs8177412 affects *GPX3* gene expression and activity, in addition to altered levels of GSH due to the AA exposure. Taken together, *GPX3* genotyping may prove as a desirable marker of the disease, aiding in identifying BEN patients more prone to develop upper urothelial tumors.

In this study several limitations are noted. One of the limitations is relatively small sample size. Due to the fact that BEN is a disease that affects only a targeted population, these results can be beneficial for a portion of this population and probably cannot be transferred to a heterogenous populations. Besides age and gender as confounding factors, there are probably more confounding factors that might affect the results. Also, the limitation is the lack of BEN patients or controls carrying all six non-risk associated genotypes (KEAP1*CC, Nrf2*CC, GSTPAB*AA, GSTPCD*CC, GPX3*TT, MDR1*CC) and all six risk-associated genotypes (KEAP1*GG, Nrf2*AA, GSTPAB*GG, GSTPCD*TT, GPX3*CC, MDR1*TT). Therefore, the combined effect and the total cumulative effect of all six genotypes could not be analyzed. Hopefully, we will continue this study with a larger cohort of participants in order to analyze combined and cumulative effects of all six genotypes.

## 5. Conclusions

Based on our findings, it may be concluded that susceptibility to BEN and its associated tumors is not related to polymorphism in regulatory antioxidant proteins Nrf2 and Keap1 or their target gene *GSTP1*. Regarding *GPX3* rs8177412 polymorphism, the gene variant that confers lower expression is associated with significant increase in upper urothelial carcinoma risk. In the view of the fact that this feature is also characteristic of urinary bladder tumors, the role of this polymorphism should be further evaluated in terms of its biomarker potential.

## Figures and Tables

**Table 1:** Demographic and clinical characteristics of BEN patients and controls.

Variable	Patients, *n* = 209	Controls, *n* = 140	*p*
Sex			
Male, *n* (%)	117 (56)	71 (51)	
Female, *n* (%)	92 (44)	69 (49)	0.146
Age (years) *	71.67 ± 6.54	70.29 ± 6.94	0.153
Smoking, *n* (%) **			
Yes	69 (33)	37 (27)	
No	137 (67)	98 (73)	0.236
Upper tract urothelial carcinoma,			
*n* (%) **			
Yes	32 (15)	//	
No	177 (85)	//	-

* mean ± SD; BMI—body mass index; ** based on available information; // not applicable.

**Table 2:** *KEAP1*, *Nrf2*, *GSTP1AB*, *GSTP1CD*, *GPX3* and *MDR1* genotype distributions in BEN patients and controls and the risk for BEN development.

Genotype	Patients, *n* (%)	Controls, *n* (%)	OR ^a^ 1 (95% CI) ^b^	*p*	OR 2 (95% CI)	*p*	OR 3 (95% CI)	*p*	OR 4 (95% CI)	*p*
*KEAP1* rs1048290										
*C/C ^c^	35 (16)	22 (16)	1.0 ^c^	-	1.0 ^c^	-	1.0 ^c^	-	1.0 ^c^	-
*C/G	87 (42)	61 (43)	0.89 (0.48–1.67)	0.732	0.86 (0.45–1.63)	0.861	0.91 (0.48 1.72)	0.791	0.90 (0.46–1.74)	0.759
*G/G	87 (42)	57 (41)	0.95 (0.51–1.8)	0.897	0.90 (0.54–2.08)	0.763	0.97 (0.51–1.84)	0.940	0.96 (0.49–1.87)	0.920
*C/G*G/G	174 (84)	118 (84)	0.92 (0.51–1.65)	0.798	0.88 (0.49–1.6)	0.688	0.94 (0.52–1.70)	0.853	0.93 (0.51–1.70)	0.834
*Nrf2* rs6721961										
*C/C ^c^	158 (76)	103 (74)	1.0 ^c^	-	1.0 ^c^	-	1.0 ^c^	-	1.0 ^c^	-
*C/A	45 (21)	34 (24)	0.86 (0.52–1.44)	0.571	0.88 (0.53–1.48)	0.648	0.86 (0.51–1.45)	0.587	0.89 (0.52–1.51)	0.671
*A/A	6 (3)	3 (2)	1.30 (0.32–0.33)	0.712	0.90 (0.19–4.17)	0.901	1.15 (0.27–4.75)	0.847	0.67 (0.14–3.21)	0.625
*C/A*A/A	51(24)	37(26)	0.88 (0.55–1.46)	0.669	0.91 (0.55–1.50)	0.723	0.89 (0.54–1.46)	0.648	0.83 (0.48–1.42)	0.608
*GSTP1 AB* rs1695										
*A/A ^c^	96 (45)	64 (46)	1.0 ^c^	-	1.0 ^c^	-	1.0 ^c^		1.0 ^c^	-
*A/G	95 (46)	67 (48)	0.94 (0.60–1.47)	0.804	0.92 (0.58–1.46)	0.737	0.91 (0.58–1.44)	0.710	0.98 (0.61–1.56)	0.930
*G/G	18 (9)	9 (6)	1.33 (0.56–3.15)	0.512	1.04 (0.42–2.57)	0.931	1.34 (0.56–3.21)	0.501	0.93 (0.36–2.24)	0.894
*A/G*G/G	113 (55)	76 (54)	0.99 (0.64–1.52)	0.968	0.94 (0.60–1.47)	0.814	0.96 (0.62–1.49)	0.887	0.97 (0.62–1.53)	0.913
*GSTP1 CD* rs1138272										
*C/C ^c^	187 (89)	129(92)	1.0 ^c^	-	1.0 ^c^	-	1.0 ^c^	-	1.0 ^c^	-
*C/T	17 (8)	11(8)	1.06 (0.48–2.35)	0.874	1.07 (0.47–2.41)	0.864	1.01 (0.45–2.26)	0.967	0.96 (0.61–1.56)	0.926
*T/T	5 (3)	0(0)	NA ^d^	NA	NA	NA	NA	NA	NA	NA
*C/T*T/T	22(11)	11 (8)	1.38 (0.64–2.94)	0.405	1.40 (0.64–3.04)	0.396	1.35 (0.63–2.90)	0.438	1.31 (0.59–2.90)	0.505
*GPX3* rs8177412										
*T/T ^c^	159(76)	112 (80)	1.0 ^c^	-	1.0 ^c^	-	1.0 ^c^	-	1.0 ^c^	-
*T/C	49 (23)	26 (19)	1.32 (0.77–2.26)	0.298	1.30 (0.75–2.25)	0.124	1.33 (0.77–2.78)	0.294	1.30 (0.74–2.25)	0.351
*C/C	1 (1)	2 (1)	0.35 (0.03–3.93)	0.397	0.37 (0.32–4.34)	0.429	0.40 (0.03–4.50)	0.459	0.45 (0.03–5.23)	0.526
*T/C*C/C	50 (24)	28 (20)	1.25 (0.74–2.12)	0.389	1.24 (0.73–2.11)	0.415	1.26 (0.75–2.14)	0.374	1.26 (0.74–2.15)	0.392
*MDR1* rs1045642										
*C/C ^c^	39 (20)	36 (26)	1.0 ^c^	-	1.0 ^c^	-	1.0 ^c^	-	1.0 ^c^	-
*C/T	100 (49)	69 (49)	1.33 (0.77–2.31)	0.297	1.31 (0.75–2.29)	0.341	1.37 (0.78–2.38)	0.263	1.35 (0.77–2.39)	0.290
*T/T	43 (31)	35 (25)	1.66 (0.90–3.06)	0.105	1.55 (0.83–2.90)	0.164	1.82 (0.97–3.40)	0.059	1.70 (0.99–3.22)	0.099
*C/T*T/T	143 (80)	104 (74)	1.44 (0.86–2.42)	0.160	1.45 (0.86–2.44)	0.159	1.51 (0.90–2.56)	0.117	1.47 (0.86–2.50)	0.149

^a^ OR—odds ratio; OR 1—crude results, without confounding factors; OR 2—with other genes as confounding factors; OR 3—with age and sex as confounding factors; OR 4—with all previously stated confounding factors; ^b^ 95% CI—95% confidence interval; *p* < 0.05 was considered statistically significant; ^c^ Referent group; ^d^ NA—not applicable (in the case of GSTP1CD rs1138272*T/T, there were no carriers in the control group (*n* = 0); therefore, the OR could not be calculated).

**Table 3:** The association of combined *KEAP1*, *Nrf2*, *GSTP1AB*, *GSTP1CD*, *GPX3* and *MDR1* genotypes with the risk of BEN development.

Genotype	BEN, *n* (%)	Controls, *n* (%)	OR 1 ^a^ (95% CI) ^b^	*p*	OR 2 (95% CI)		*p*
*GPX3* rs8177412+ *MDR1* rs1045642							
GPX3*TT/MDR1*CC	33 (16)	31 (22)	1.0 ^c^	-	1.0 ^c^		-
GPX3*TT/MDR1*CT*TT	120( 60)	81 (58)	1.39 (0.79–2.45)	0.252	1.46 (0.82–2.60)		0.193
GPX3*CC*TC/MDR1*CC	6 (3)	5 (4)	1.12 (0.31–4.07)	0.855	1.16 (0.31–4.22)		0.820
GPX3*CC*TC/MDR1*CT*TT	43 (21)	23 (16)	1.75 (0.86–3.55)	0.117	1.86 (0.91–3.80)		0.087
*Nrf2* rs6721961+ *KEAP1* rs1048290							
Nrf2*CC/KEAP1*CC	25 (12)	18 (13)	1.0 ^c^	-	1.0 ^c^		-
Nrf2*CC/KEAP1*CG*GG	10 (5)	4 (3)	1.80 (0.48–6.62)	0.379	1.91 (0.51–7.14)		0.335
Nrf2*CA*AA/KEAP1*CC	133 (63)	85 (61)	1.12 (0.58–2.18)	0.725	1.17 (0.60–2.30)		0.634
Nrf2*CA*AA/KEAP1*CG*GG	41(20)	33 (23)	0.89 (0.41–1.91)	0.774	0.91 (0.42–1.96)		0.818
*Nrf2* rs6721961+ *MDR1* rs1045642							
Nrf2*CC/MDR1*CC	31 (15)	28 (20)	1.0 ^c^	-	1.0 ^c^		-
Nrf2*CC/MDR1*CT*TT	121 (60)	75 (53)	1.45 (0.81–2.62)	0.208	1.52 (0.83–2.75)		0.167
Nrf2*CA*AA/MDR1*CC	8 (4)	8 (6)	0.90 (0.29–2.72)	0.857	0.87 (0.28–2.68)		0.811
Nrf2*CA*AA/MDR1*CT*TT	42 (21)	29 (21)	1.30 (0.65–2.62)	0.450	1.35 (0.67–2.74)		0.396
*Nrf2* rs6721961+ *GSTP1AB rs1695*							
Nrf2*CC/GSTP1AB*AA	70 (34)	43 (31)	1.0 ^c^	-	1.0 ^c^		-
Nrf2*CC/GSTP1AB*AG*GG	88 (42)	60 (43)	0.90 (0.54–1.48)	0.684	0.87 (0.52–1.44)		0.599
Nrf2*CA*AA/GSTP1AB*AA	26 (12)	21 (15)	0.76 (0.38–1.51)	0.436	0.74 (0.37–1.48)		0.399
Nrf2*CA*AA/GSTP1AB*AG*GG	25 (12)	16 (11)	0.96 (0.46–1.99)	0.913	0.93 (0.44–1.95)		0.856
*Nrf2* rs*6721961*+ *GSTP1CD* rs1138272							
Nrf2*CC/GSTPD*CC	141 (68)	95 (68)	1.0 ^c^	-	1.0 ^c^		-
Nrf2*CC/GSTP1CD*CT*TT	17 (8)	8 (6)	1.43 (0.59–3.45)	0.424	1.36 (0.56–3.31)		0.494
Nrf2*CA*AA/GSTP1CD*CC	46 (22)	34 (24)	0.91 (0.54–1.52)	0.724	0.89 (0.53–1.50)		0.675
Nrf2*CA*AA/GSTP1CD*CT*TT	5 (2)	3 (2)	1.12 (0.26–4.81)	0.876	1.18 (0.27–5.14)		0.825

^a^ OR—odds ratio; OR 1—crude results, without confounding factors; OR 2—with age and gender as confounding factors; ^b^ 95% CI—95% confidence interval; *p* < 0.05 was considered statistically significant; ^c^—referent group.

**Table 4:** The association of combined *KEAP1*, *Nrf2*, *GSTP1AB*, *GSTP1CD*, *GPX3* and *MDR1* genotypes with the risk of BEN development in females.

Genotype	BEN, *n* (%)	Controls, *n* (%)	OR 1 ^a^ (95% CI) ^b^	*p*	OR 2 (95% CI)	*p*
*GPX3* rs8177412+ *MDR1* rs1045642						
GPX3*TT/MDR1*CC	13 (14)	17 (24)	1.0 ^c^	-	1.0 ^c^	-
GPX3*TT/MDR1*CT*TT	51 (57)	43 (60)	1.55 (0.67–3.55)	0.299	1.85 (0.78–4.40)	0.163
GPX3*CC*TC/MDR1*CC	3 (3)	2 (3)	1.96 (0.28–13.50)	0.494	2.11 (0.30–14.74)	0.451
GPX3*CC*TC/MDR1*CT*TT	23 (26)	9 (13)	3.34 (1.16–9.60)	0.025 *	3.79 (1.27–11.24)	0.016 *
*Nrf2* rs6721961+ *KEAP1* rs1048290						
Nrf2*CC/KEAP1*CC	6 (7)	9 (13)	1.0 ^c^	-	10 ^c^	-
Nrf2*CC/KEAP1*CG*GG	61 (66)	48 (68)	1.80 (0.48–6.62)	0.379	1.87 (0.61–5.71)	0.269
Nrf2*CA*AA/KEAP1*CC	5 (5)	2 (2)	1.12 (0.58–2.18)	0.725	3.65 (0.51–25.73)	0.194
Nrf2*CA*AA/KEAP1*CG*GG	20(22)	12 (17)	0.89 (0.41–1.91)	0.774	2.35 (0.65–8.40)	0.187
*Nrf2* rs6721961+ *MDR1* rs1045642						
Nrf2*CC/MDR1*CC	13 (15)	18 (25)	1.0 ^c^	-	1.0 ^c^	-
Nrf2*CC/MDR1*CT*TT	52 (57)	39 (55)	1.84 (0.80–4.21)	0.145	2.19 (0.92–5.18)	0.074
Nrf2*CA*AA/MDR1*CC	3 (3)	1 (1)	4.15 (0.38–44.50)	0.240	4.07 (0.36–44.97)	0.252
Nrf2*CA*AA/MDR1*CT*TT	22 (25)	13 (19)	2.34 (0.87–6.30)	0.092	2.60 (0.94–7.18)	0.065
*Nrf2* rs6721961+ *GSTP1AB rs1695*						
Nrf2*CC/GSTP1AB*AA	29 (32)	27 (38)	1.0 ^c^	-	1.0 ^c^	-
Nrf2*CC/GSTP1AB*AG*GG	38 (41)	30 (42)	1.17 (0.58–2.39)	0.649	1.05 (0.50–2.18)	0.886
Nrf2*CA*AA/GSTP1AB*AA	12 (13)	8 (11)	1.39 (0.49–3.93)	0.528	1.30 (0.45–3.73)	0.620
Nrf2*CA*AA/GSTP1AB*AG*GG	13 (14)	6 (9)	2.07 (0.67–6.06)	0.211	1.76 (0.57–5.38)	0.319
*Nrf2* rs*6721961*+ *GSTP1CD* rs1138272						
Nrf2*CC/GSTP1CD*CC	63 (69)	53 (75)	1.0 ^c^	-	1.0 ^c^	-
Nrf2*CC/GSTP1CD*CT*TT	4 (4)	4 (6)	1.05 (0.50–2.18)	0.886	0.70 (0.16–3.09)	0.648
Nrf2*CA*AA/GSTP1CD*CC	22 (24)	13 (18)	1.30 (0.45–3.73)	0.620	1.33 (0.60–2.92)	0.477
Nrf2*CA*AA/GSTP1CD*CT*TT	3 (3)	1 (1)	1.76 (0.57–5.38)	0.319	2.75 (0.27–27.82)	0.389

^a^ OR—odds ratio; OR 1—crude results, without confounding factors; OR 2—with age as confounding factor; ^b^ 95% CI—95% confidence interval; *p* < 0.05 was considered statistically significant; ^c^—referent group.

**Table 5:** The association of combined *KEAP1*, *Nrf2*, *GSTP1AB*, *GSTP1CD*, *GPX3* and *MDR1* genotypes with the risk of BEN development in males.

Genotype	BEN, *n* (%)	Controls, *n* (%)	OR 1 ^a^ (95% CI) ^b^	*p*	OR 2 (95% CI)	*p*
*GPX3* rs8177412+ *MDR1* rs1045642						
GPX3*TT/MDR1*CC	20 (18)	14 (20)	1.0 ^c^	-	1.0 ^c^	-
GPX3*TT/MDR1*CT*TT	69(61)	38 (55)	1.27 (0.57–2.79)	0.552	1.27 (0.57–2.81)	0.547
GPX3*CC*TC/MDR1*CC	3 (3)	3 (4)	0.70 (0.12–3.98)	0.688	0.70 (0.12–4.05)	0.699
GPX3*CC*TC/MDR1*CT*TT	20 (18)	14 (21)	1.00 (0.38–2.67)	1.000	1.02 (0.38–2.691)	0.996
*Nrf2* rs6721961+ *KEAP1* rs1048290						
Nrf2*CC/KEAP1*CC	19 (16)	9 (13)	1.0 ^c^	-	1.0 ^c^	-
Nrf2*CC/KEAP1*CG*GG	72 (62)	37 (54)	0.85 (0.38–2.23)	0.857	0.92 (0.38–2.24)	0.861
Nrf2*CA*AA/KEAP1*CC	5 (4)	2 (3)	1.18 (0.19–7.32)	0.856	1.20 (0.19–7.45)	0.842
Nrf2*CA*AA/KEAP1*CG*GG	21(18)	21 (30)	0.47 (0.17–1.28)	0.142	0.47 (0.17–1.30)	0.148
*Nrf2* rs6721961+ *MDR1* rs1045642						
Nrf2*CC/MDR1*CC	18 (16)	10 (15)	1.0 ^c^	-	1.0 ^c^	-
Nrf2*CC/MDR1*CT*TT	69 (62)	36 (52)	1.06 (0.44–2.54)	0.888	1.07 (0.44–2.57)	0.872
Nrf2*CA*AA/MDR1*CC	5 (4)	7 (10)	0.39 (0.09–1.58)	0.190	0.40 (0.10–1.61)	0.199
Nrf2*CA*AA/MDR1*CT*TT	20 (18)	16 (23)	0.69 (0.25–1.91)	0.481	0.70 (0.25–1.95)	0.501
*Nrf2* rs6721961+ *GSTP1AB rs1695*						
Nrf2*CC/GSTP1AB*AA	41 (35)	16 (23)	1.0 ^c^	-	1.0 ^c^	-
Nrf2*CC/GSTP1AB*AG*GG	50 (43)	30 (43)	0.65 (0.32–1.35)	0.251	0.65 (0.31–1.37)	0.264
Nrf2*CA*AA/GSTP1AB*AA	14 (12)	13 (19)	0.42 (0.16–1.08)	0.074	0.42 (0.16–1.10)	0.079
Nrf2*CA*AA/GSTP1AB*AG*GG	12 (10)	10 (15)	0.46 (0.16–1.29)	0.144	0.47 (0.17–1.32)	0.155
*Nrf2* rs*6721961*+ *GSTP1CD* rs1138272						
Nrf2*CC/GSTP1CD*CC	78 (67)	42 (61)	1.0 ^c^	-	1.0 ^c^	-
Nrf2*CC/GSTP1CD*CT*TT	13 (11)	4 (6)	1.75 (0.53–5.75)	0.353	1.78 (0.54–5.82)	0.339
Nrf2*CA*AA/GSTP1CD*CC	24 (21)	21 (30)	0.61 (0.30–1.23)	0.171	0.62 (0.31–1.25)	0.184
Nrf2*CA*AA/GSTP1CD*CT*TT	2 (1)	2 (3)	0.53 (0.07–3.96)	0.543	0.54 (0.07–4.03)	0.553

^a^ OR—odds ratio; OR 1—crude results, without confounding factors; OR 2—with age as confounding factor; ^b^ 95% CI—95% confidence interval; *p* < 0.05 was considered statistically significant; ^c^—referent group.

**Table 6:** Effects of GSTP1 rs1695 and rs1138272 polymorphisms.

L1	L2	D′	LOD	r^2^	95% CI
*GSTP1AB*rs1695	*GSTP1CD*rs1138272	0.647	4.63	0.051	0.41–0.80

D′—the value of D prime between the two loci; LOD—the log of the likelihood odds ratio, a measure of confidence in the value of D′; r^2^—the correlation coefficient between the two loci; 95% CI—95% confidence lower bound on D′.

**Table 7:** Haplotype analysis of GSTP1 rs1695 and rs1138272 polymorphisms in patients with BEN.

	Genotype		Haplotype Frequencies			
	rs1695	rs1138272	BEN, %	Controls, %	OR (95% CI) ^a^	*p*
*GSTP1A* ^d^	*A	*C	68	70	1.00 ^c^	
*GSTP1B* ^e^	*G	*C	26	27	1.01 (0.70–1.45)	0.970
*GSTP1C* ^f^	*G	*T	5	3	1.64 (0.75–3.58)	0.210
*GSTP1D* ^g^	*A	*T	1	0	NA ^b^	NA

^a^ OR: odds ratio adjusted for age, gender, BMI, pack-years and hypertension; CI: confidence interval; ^c^ reference group; ^b^ NA: not applicable; ^d^—GSTP1A genotype consisting of Ile105 and Ala114; ^e^—GSTP1B genotype consisting of Val105 and Ala114; ^f^—GSTP1C genotype consisting of Val105 and Val114; ^g^—GSTP1D genotype consisting of Ile105 and Val114.

**Table 8:** The cumulative association of polymorphisms encoding for regulatory and catalytic antioxidant proteins with the risk for BEN development.

Genotype	BEN, *n* (%)	Controls, *n* (%)	OR 1 ^a^ (95% CI) ^b^	*p*
1 ^c^	20 (10)	16 (11)	1.00 ^c^	-
2	65 (32)	45 (32)	1.32 (0.57–2.68)	0.591
3	71 (35)	48 (34)	1.04 (0.45–2.43)	0.916
4	33 (16)	26 (18)	2.32 (0.62–8.71)	0.210
5	12 (6)	4 (3)	3.04 (0.74–12.3)	0.120

^a^ OR—odds ratio; ^b^ 95% CI—95%confidence interval; *p* < 0.05 was considered statistically significant; ^c^—Reference group. 1,2,3,4,5: The number of the present risk-associated genotypes, i.e., either one risk-associated genotype, or two risk-associated genotypes, or three risk-associated genotypes, or four risk-associated genotypes or five risk-associated genotypes. There was no recruited cases or controls carrying all six referent genotypes (KEAP1*CC, Nrf2*CC, GSTPAB*AA, GSTPCD*CC, GPX3*TT, MDR1*CC) or all six risk-associated genotypes (KEAP1*GG, Nrf2*AA, GSTPAB*GG, GSTPCD*TT, GPX3*CC, MDR1*TT).

**Table 9:** Association of polymorphisms encoding regulatory and catalytic antioxidant proteins with transitional cell carcinoma (in patients with Balkan endemic nephropathy).

Genotype	BEN with Tumors, *n = 32* *n* (%)	BEN without Tumors, *n = 177* *n* (%)	OR^a^ 1 (95% CI) ^b^	*p*	OR 2 (95% CI)	*p*
*KEAP1* rs1048290						
*CC	4 (12)	31 (18)	1.0 ^c^	-	1.0 ^c^	-
*CG*GG	28 (88)	146 (82)	1.48 (0.48–4.52)	0.487	0.84 (0.34–2.02)	0.718
*Nrf2* rs6721961						
*CC	25 (78)	133 (75)	1.0 ^c^	-	1.0 ^c^	-
*CA*AA	7 (22)	44 (25)	0.84 (0.34–2.09)	0.718	0.83 (0.33–2.07)	0.693
*GSTP1AB* rs1695						
*AA	12 (38)	84 (47)	1.0 ^c^	-	1.0 ^c^	-
*AG*GG	20 (62)	93 (53)	1.30 (0.69–3.26)	0.500	1.41 (0.64–3.10)	0.382
*GSTP1CD* rs1138272						
*CC	27 (84)	160 (90)	1.0 ^c^	-	1.0 ^c^	-
*CT*TT	5 (16)	17 (10)	1.71 (0.59–5.11)	0.312	1.84 (0.61–5.53)	0.275
*GPX3* rs8177412						
*TT	12 (38)	147 (83)	1.0 ^c^	-	1.0 ^c^	-
*CC*TC	20 (62)	30 (17)	8.16 (3.60–18.40)	0.001 *	8.48 (3.60–19.30)	0.001 *

^a^ OR—odds ratio; OR 1—crude results, without confounding factors; OR 2—with age and gender as confounding factors; ^b^ 95% CI—95% confidence interval; *p* < 0.05 was considered statistically significant; ^c^—referent group.

## Data Availability

The data supporting reported results can be obtained upon request in the form of datasets available at The Faculty of Medicine, University of Banja Luka, the Republic of Srpska, Bosnia and Herzegovina and Institute of Medical and Clinical Biochemistry, Faculty of Medicine, University of Belgrade, Serbia.

## Supplementary Materials

The following supporting information can be downloaded at: https://www.mdpi.com/article/10.3390/medicina59081421/s1, Table S1. *KEAP1*, *Nrf2*, *GSTP1AB*, *GSTP1CD*, *GPX3* and *MDR1* genotype distributions in female BEN patients and controls and the risk for BEN development and Table S2. *KEAP1*, *Nrf2*, *GSTP1AB*, *GSTP1CD*, *GPX3* and *MDR1* genotype distributions and the risk for BEN development in male BEN patients and controls.
