# Supplementary material for: GPX3 rs8177412 Polymorphism Modifies Risk of Upper Urothelial Tumors in Patients with Balkan Endemic Nephropathy
Source: Medicina (Kaunas). 2023 Aug 4;59(8):1421. doi: 10.3390/medicina59081421 (PMC10456338; doi:10.3390/medicina59081421)
Supplement: Supplementary file 1 [file medicina-59-01421-s001.zip › medicina-2524832-supplementary.pdf]

Table S1. *KEAP1, Nrf2, GSTP1AB, GSTP1CD, GPX3* and *MDR1* genotype distributions in female BEN patients and controls, and the risk for BEN development

**Genotype**	**Patients, *n* (%)**	**Controls, *n* (%)**	**OR 1[a] (95% CI)**	***p***	**OR 2 (95% CI)**	***p***	**OR 3 (95% CI)**	***p***	**OR 4 (95% CI)**	***p***
KEAP1 rs1048290										
**C/C*[c]	11 (12)	11 (15)	1.0	-	1.0	-	1.0		1.0	-
**C/G*G/G*	81 (88)	60 (85)	1.35 (0.54-3.32)	0.513	1.49 (0.58-3.78)	0.399	1.32 (0.53-3.28)	0.549	1.43 (0.55-3.71)	0.453
Nrf2 rs6721961										
**C/C*[c]	67 (73)	57 (80)	1.0	-	1.0	-	1.0		1.0	-
**C/A*A/A*	25 (27)	14 (20)	1.51 (0.72-3.19)	0.270	1.47 (0.68-3.18)	0.323	1.46 (0.68-3.10)	0.323	1.39 (0.63-3.05)	0.406
GSTP1AB rs1695										
**A/A*[c]	41 (45)	35 (49)	1.0	-	1.0	-	1.0		1.0	-
**A/G*G/G*	51 (55)	36 (51)	1.20 (0.65-2.25)	0.548	1.27 (0.66-2.45)	0.461	1.09 (0.57-2.06)	0.781	1.15 (0.59-2.25)	0.667
GSTP1CD rs1138272										
**C/C*[c]	85 (92)	66 (93)	1.0	-	1.0	-	1.0		1.0	-
**C/T*T/T*	7 (8)	5 (7)	1.08 (0.33-3.58)	0.891	0.96 (0.27-3.36)	0.961	1.02 (0.30-3.45)	0.970	1.27 (0.55-2.93)	0.931
GPX3 rs8177412										
**T/T*[c]	66 (72)	60 (85)	1.0	-	1.0	-	1.0		1.0	-
**T/C*C/C*	26 (28)	11 (15)	2.14 (0.97-4.72)	0.057	2.15 (0.96-4.81)	0.062	2.09 (0.94-4.64)	0.068	2.09 (0.92-4.72)	0.075
MDR1 rs1045642										
**C/C*[c]	16 (18)	19 (27)	1.0	-	1.0	-	1.0		1.0	-
**C/T*T/T*	74 (82)	52 (73)	1.69 (0.79-3.13)	0.172	1.51 (0.69-3.29)	0.296	1.97 (0.89-4.32)	0.090	1.75 (0.78-3.93)	0.169

[a]OR—odds ratio; OR 1—crude results, without confounding factors; OR 2—with other two genes as confounding factors; OR 3—with age as confounding factor; OR 4—with all previously stated confounding factors; 95%CI—95% confidence interval; $p < 0.05$ was considered statistically significant;[c] referent group.

Table S2. *KEAP1, Nrf2, GSTP1AB, GSTP1CD, GPX3* and *MDR1* genotype distributions in male BEN patients and controls, and the risk for BEN development

**Genotype**	**Patients, *n* (%)**	**Controls, *n* (%)**	**OR 1[a] (95% CI)**	***p***	**OR 2 (95% CI)**	***p***	**OR 3 (95% CI)**	***p***	**OR 4 (95% CI)**	***p***
KEAP1 rs1048290										
**C/C*[c]	24 (20)	11 (16)	1.0	-	1.0	-	1.0	-	1.0	-
**C/G*G/G*	93 (80)	58 (84)	0.73 (0.33-1.61)	0.442	0.77 (0.34-1.74)	0.531	0.73 (0.33-1.61)	0.444	0.76 (0.34-1.73)	0.528
Nrf2 rs6721961										
**C/C*[c]	91 (78)	46 (67)	1.0	-	1.0	-	1.0	-	1.0	-
**C/A*A/A*	26 (22)	23 (33)	0.57 (0.29-1.10)	0.098	0.57 (0.29-1.13)	0.110	1.01 (0.96-1.05)	0.175	0.58 (0.29-1.14)	0.118
GSTP1AB rs1695										
**A/A*[c]	55 (47)	29 (42)	1.0	-	1.0	-	1.0	-	1.0	-
**A/G*G/G*	62 (53)	40 (58)	0.81 (0.44-1.49)	0.510	0.73 (0.38-1.39)	0.348	0.82 (0.45-1.50)	0.531	0.74 (0.39-1.41)	0.370
GSTP1CD rs1138272										
**C/C*[c]	102 (87)	63 (92)	1.0	-	1.0	-	1.0	-	1.0	-
**C/T*T/T*	15 (13)	6 (8)	1.54 (0.56-4.18)	0.393	1.66 (0.58-4.81)	0.342	1.56 (0.57-4.26)	0.377	1.67 (0.58-4.81)	0.340
GPX3 rs8177412										
**T/T*[c]	93 (80)	52 (75)	1.0	-	1.0	-	1.0	-	1.0	-
**T/C*C/C*	24 (20)	17 (25)	0.78 (0.38-1.60)	0.513	0.80 (0.38-1.66)	0.554	0.79 (0.39-1.62)	0.536	0.81 (0.39-1.70)	0.587
MDR1 rs1045642										
**C/C*[c]	23 (20)	17 (25)	1.0	-	1.0	-	1.0	-	1.0	-
**C/T*T/T*	89 (80)	52 (75)	1.26 (0.61-2.58)	0.519	1.16 (0.55-2.44)	0.679	1.27 (0.62-2.60)	0.509	1.17 (0.56-2.45)	0.670

[a]OR—odds ratio; OR 1—crude results, without confounding factors; OR 2—with other two genes as confounding factors; OR 3—with age as confounding factor; OR 4—with all previously stated confounding factors; 95%CI—95% confidence interval; $p < 0.05$ was considered statistically significant;[c] referent group.
